# Integrated Care for Atrial Fibrillation Using the ABC Pathway in the Prospective APHRS-AF Registry

**DOI:** 10.1016/j.jacasi.2023.04.008

**Published:** 2023-06-27

**Authors:** Tommaso Bucci, Marco Proietti, Alena Shantsila, Giulio Francesco Romiti, Wee-Siong Teo, Hyung-Wook Park, Wataru Shimizu, Hung-Fat Tse, Gregory Y.H. Lip, Tze-Fan Chao, APHRS-AF Registry Investigators

**Affiliations:** a Liverpool Centre of Cardiovascular Science at University of Liverpool, Liverpool John Moores University and Liverpool Heart and Chest Hospital, Liverpool, United Kingdom; b Department of General and Specialized Surgery, Sapienza University of Rome, Rome, Italy; c Division of Subacute Care, IRCCS Istituti Clinici Scientifici Maugeri, Milan, Italy; d Department of Clinical Sciences and Community Health, University of Milan, Milan, Italy; e Department of Translational and Precision Medicine, Sapienza University of Rome, Rome, Italy; f Department of Cardiology, National Heart Centre, Singapore; g Department of Cardiovascular Medicine, Chonnam National University Hospital, Gwangju, Korea; h Department of Cardiovascular Medicine, Nippon Medical School, Tokyo, Japan; i Division of Cardiology, Department of Medicine, School of Clinical Medicine, Queen Mary Hospital, the University of Hong Kong, Hong Kong SAR, China; j Danish Center for Clinical Health Services Research, Department of Clinical Medicine, Aalborg University, Denmark; k Institute of Clinical Medicine and Cardiovascular Research Center, National Yang Ming Chiao Tung University, Taipei, Taiwan; l Division of Cardiology, Department of Medicine, Taipei Veterans General Hospital, Taipei, Taiwan

**Keywords:** atrial fibrillation, integrated care, quality indicators

## Abstract

**Background:**

The Atrial Fibrillation Better Care (ABC) has been proposed as an integrated approach to improve management in patients with atrial fibrillation (AF), based on 3 pillars: “A” Avoid stroke with Anticoagulation; “B” Better symptoms control; “C” Cardiovascular risk-factor and comorbidities management.

**Objectives:**

This study sought to investigate the association with outcomes of ABC adherence in the prospective multinational Asia-Pacific Heart Rhythm Society (APHRS) Atrial Fibrillation registry.

**Method:**

Cox-regression analyses adjusted for age, sex, CHA_2_DS_2_-VASc score, paroxysmal AF, chronic obstructive pulmonary disease, chronic kidney disease, cancer, dyslipidemia, and dementia were performed to investigate the association with outcomes. Primary outcome was a composite of all-cause death, any thromboembolic events, acute coronary syndrome or percutaneous interventional procedures, and advancing heart failure.

**Results:**

Of the 4,013 included patients with AF (mean age 68 ± 12 years; 34.4% female); 38.6% were adherent to all 3 main ABC pillars. After 1 year of follow-up, adherence to the ABC pathway was associated with a low incidence of composite outcome (4.0% vs 8.5%, *P* < 0.001), all-cause and cardiovascular death, and advancing heart failure. On Cox regression analysis, ABC adherence was associated with a lower risk of primary outcome (HR: 0.72; 95% CI: 0.53-0.97), with risk reduction progressively higher with a higher number of ABC criteria attained. No significant interaction in the association was seen according to the different geographic areas (*P*_int_ = 0.217).

**Conclusions:**

In a large contemporary cohort of Asian patients with AF, adherence to ABC pathway was associated with a reduction of the risk for adverse outcomes. (Clinical Survey on the Stroke Prevention in Atrial Fibrillation in Asia (AF-Registry; NCT04807049)

Atrial fibrillation (AF) is the most common arrhythmia worldwide and is associated with increased morbidity and mortality.^1^ However, despite oral anticoagulation (OAC) with vitamin-K antagonists (VKAs) or direct-acting oral anticoagulants (DOACs) showing a significant survival improvement in patients with AF,^2^ almost 70% of the causes of cardiovascular deaths in AF are not linked to thromboembolic events (TEs).^3^^,^^4^ Indeed, in the ROCKET-AF (Rivaroxaban Once Daily Oral Direct Factor Xa Inhibition Compared with Vitamin K Antagonism for Prevention of Stroke and Embolism Trial in Atrial Fibrillation) study on 14,171 patients with AF, after a median follow-up of 1.9 years, <10% of the cardiovascular deaths were associated with ischemic stroke or systemic embolism,^3^ whereas, in a French cohort of 8,962 patients with AF, after a median follow-up of 1.3 years, only 7% of deaths were caused by stroke.^4^ These was further confirmed in 4,664 Asian patients with AF enrolled in the Asia-Pacific Heart Rhythm Society (APHRS) AF registry in which, after 1 year of follow-up, ischemic stroke was associated only with 1% of the reported deaths.^5^

AF often coexists with other cardiovascular comorbidities, such as arterial hypertension, heart failure (HF), diabetes mellitus, peripheral (PAD), and coronary artery disease (CAD),^6^ as well as with an overall high burden of multimorbidity.^7^ The presence of multiple comorbidities in patients with AF requires more comprehensive clinical management not only based on OAC therapy but also on the optimal control of all the cardiovascular risk factors and comorbidities.^8^ Growing evidence shows that a holistic-care approach effectively reduces the residual risk of death and cardiovascular events (CVEs) in different AF populations.^9^, ^10^, ^11^, ^12^, ^13^, ^14^, ^15^, ^16^ The ABC (Atrial fibrillation Better Care) pathway was proposed to help facilitate holistic or integrated care management for patients with AF.^2^ The ABC pathway has 3 main pillars: “A” for avoiding stroke with appropriate prescription of OAC; “B” refers to better symptoms optimization with patient-centered symptom-directed decisions on rate and rhythm control; and “C” for optimal management of cardiovascular and management of noncardiovascular risk factors and comorbidities, including lifestyle factors.^2^ Despite several studies derived from Western populations that have already demonstrated its usefulness in reducing the risk of CVEs in patients with AF, there are relatively few prospective data on the management and treatment of patients with AF with the ABC pathway in Asian-Pacific countries.^11^, ^12^, ^13^

Since 2015, in collaboration with the European Society of Cardiology (ESC), the APHRS created the first pan-Asian prospective AF registry, enrolling patients from 5 major Asian countries (Hong Kong, South Korea, Japan, Singapore, and Taiwan) to systematically collect contemporary data regarding the management and treatment of AF.^17^

In the present study, we evaluated if clinical management adherent to the ABC pathway would be associated with a reduction in adverse outcomes in this large Asian-Pacific prospective cohort of patients with AF.

## Methods

The study protocol for patients’ enrolment and data collection was similar to the ESC European Heart Rhythm Association (EHRA) EURObservational Research Programme in AF General Long-Term Registry, as reported previously.^17^ In brief, the Registry was started in 2015, and the enrollment was finished in 2017. The population comprised consecutive inpatients and outpatients with AF who had undergone cardiology examinations in tertiary and general hospitals in 5 Asian-Pacific countries (Hong Kong, South Korea, Japan, Singapore, and Taiwan). All eligible patients had electrocardiograms (ECGs) documenting AF within 12 months before the enrollment visit and signed a written informed consents according to the local regulations. After the baseline clinical assessment, 1-year follow-up was performed by the local investigators. The study protocol was approved by the local ethics committee and was registered on ClinicalTrials.gov (NCT04807049).

### Clinical scores and evaluation of ABC pathway adherence

The CHA_2_DS_2_-VASc score was calculated as follows: congestive HF (1 point); hypertension (1 point); age 65 to 74 (1 point) and >75 years (2 points); diabetes (1 point); stroke (2 points); vascular disease (1 point); and female sex category (1 point).^18^

HAS-BLED score was calculated as follows: uncontrolled hypertension (1 point), abnormal renal or liver function (defined as dialysis, renal transplant, serum creatinine >200 mmol/L for the former and liver cirrhosis, bilirubin >2 × upper limit of normal, aspartate transaminase/alanine transaminase/alkaline phosphatase [AST/ALT/ALP] >3 x upper limit of normal for the latter, 1 point each); history of stroke (1 point); history of bleeding (1 point); labile international normalized ratio (INR) (1 point); age >65 years (1 point); and drugs (eg, aspirin or nonsteroidal anti-inflammatory drugs or alcohol) (1 point).^19^

Classification of AF-related symptoms was performed according to the European Heart Rhythm Association AF symptom classification (EHRA score)^20^ as follows: EHRA I, no symptoms; EHRA II, mild symptoms (normal daily activity not affected); EHRA III, severe symptoms (normal daily activity affected); EHRA IV, disabling symptoms (normal daily activity discontinued).

EHRA score was determined by recruiting sites and considers symptoms attributable to AF and reverse or reduce upon restoration of sinus rhythm or with effective rate control.

Adherence to the ABC pathway was evaluated according to a previous analysis performed in the ESC-EHRA EORP-AF General Long-Term Registry^16^ as follows:

#### “A” criterion

The patient was considered compliant for this criterion if properly prescribed with OAC according to the CHA_2_DS_2_-VASc score. OAC was considered as optimal treatment in male patients with CHA_2_DS_2_-VASc ≥1 or female patients with CHA_2_DS_2_-VASc ≥2; patients not qualifying for OAC therapy (CHA_2_DS_2_-VASc 0 in male patients or 1 in female patients) and not treated with OAC, also qualified for the "A" criterion. The OAC indication for the “A” criterion was made according to the 2016 AF guidelines used during the enrollment period (2015 to 2017).^21^

#### “B” criterion

Any patient with an EHRA score of I (no symptoms) or II (mild symptoms not affecting daily life) qualified for this criterion.

#### “C” criterion

We considered the following comorbidities associated with AF: hypertension, CAD, PAD, HF, stroke or transient ischemic attack (TIA), and diabetes mellitus. A patient was considered adherent to the "C’" criterion when having all the present comorbidities treated according to the current clinical guidelines. Optimal medical treatment was defined as follows: 1) for hypertension, we considered controlled blood pressure if <140/90 mm Hg at baseline; 2) for CAD, treatment with angiotensin-converting enzyme inhibitors (ACEI)-angiotensin receptor blockers (ARBs), beta blockers, and statins; 3) for PAD, treatment with statins; 4) for previous stroke and TIA, treatment with statins; 5) for HF, we considered treatment with ACEI-ARBs and beta blockers; and 6) for diabetes mellitus, treatment with insulin or oral antidiabetic agents. All patients with ≥1 clinical condition not properly treated were considered to be “C”" criterion nonadherent. Patients were considered treated as adherent to the ABC pathway if they were adherent to all 3 criteria. We also considered adherence to only 0, 1, 2, or 3 ABC pathway criteria. All patients with at least 1 ABC criterion not attained were considered to be ABC nonadherent.

### Study outcomes

Adverse outcomes were registered after 1 year of observation. The primary endpoint of the study was a composite outcome of any TE, new or worsening of a pre-existent HF (ie, advancing HF), acute coronary syndrome (ACS) or significant CAD requiring percutaneous coronary intervention (ACS or percutaneous coronary intervention [PCI]) and all-cause death. Secondary outcomes were the risk of each component of the composite outcome, cardiovascular death, and major bleeding.

All-cause death was defined as death due to cardiovascular, noncardiovascular, or unknown causes. Cardiovascular death was defined as death caused by cardiac (ACS, HF, arrhythmia, cardiac perforation, tamponade, or other unspecified cardiac causes) or vascular (ischemic stroke, hemorrhagic stroke, systemic hemorrhages, peripheral embolism, and pulmonary embolism) events.

### Statistical analysis

Categorical variables were reported as counts and percentages. Continuous variables were expressed as mean ± SD and compared by Student’s *t-*test. The chi-square test was used to compare proportions, reported as numbers and percentages. Normal distribution was assessed by the Kolmogorov-Smirnov test. The incidence rate of adverse outcomes was calculated as the number of events to total person-years ratio and reported as incidence for 100 persons per year with relative 95% CI. Cox proportional hazards regression time to the first event analysis was used to calculate the unadjusted and adjusted relative HRs and 95% CI of primary and secondary outcomes. The 1-year risks of composite adverse events were investigated and compared in ABC adherent vs ABC nonadherent groups and among patients with different numbers of ABC criteria attained. The relationship between the risk of composite outcome and the number of ABC criteria attained was investigated using 2 other models: In the first, the reference was the group of patients who did not attain any criteria (Model A); in the second, the reference was the group of patients attaining 0 or 1 criterion (Model B).

In addition, a sensitivity analysis was performed only in patients with CHA_2_DS_2_-VASc ≥2 to exclude possible bias derived from considering the ABC-adherent group patients who do not require OAC or with no concomitant comorbidities. All the multivariable Cox regression analyses were adjusted for the following covariates: age, female sex, CHA_2_DS_2_-VASc score, paroxysmal AF, chronic obstructive pulmonary disease, chronic kidney disease, cancer, dyslipidemia, and dementia. Proportional hazard assumptions were checked with the Schoenfeld residuals test. All tests were 2-tailed, and analyses were performed using computer software packages (SPSS-25.0, SPSS Inc). A *P* value <0.05 was considered to be statistically significant.

## Results

**Figure 1:**
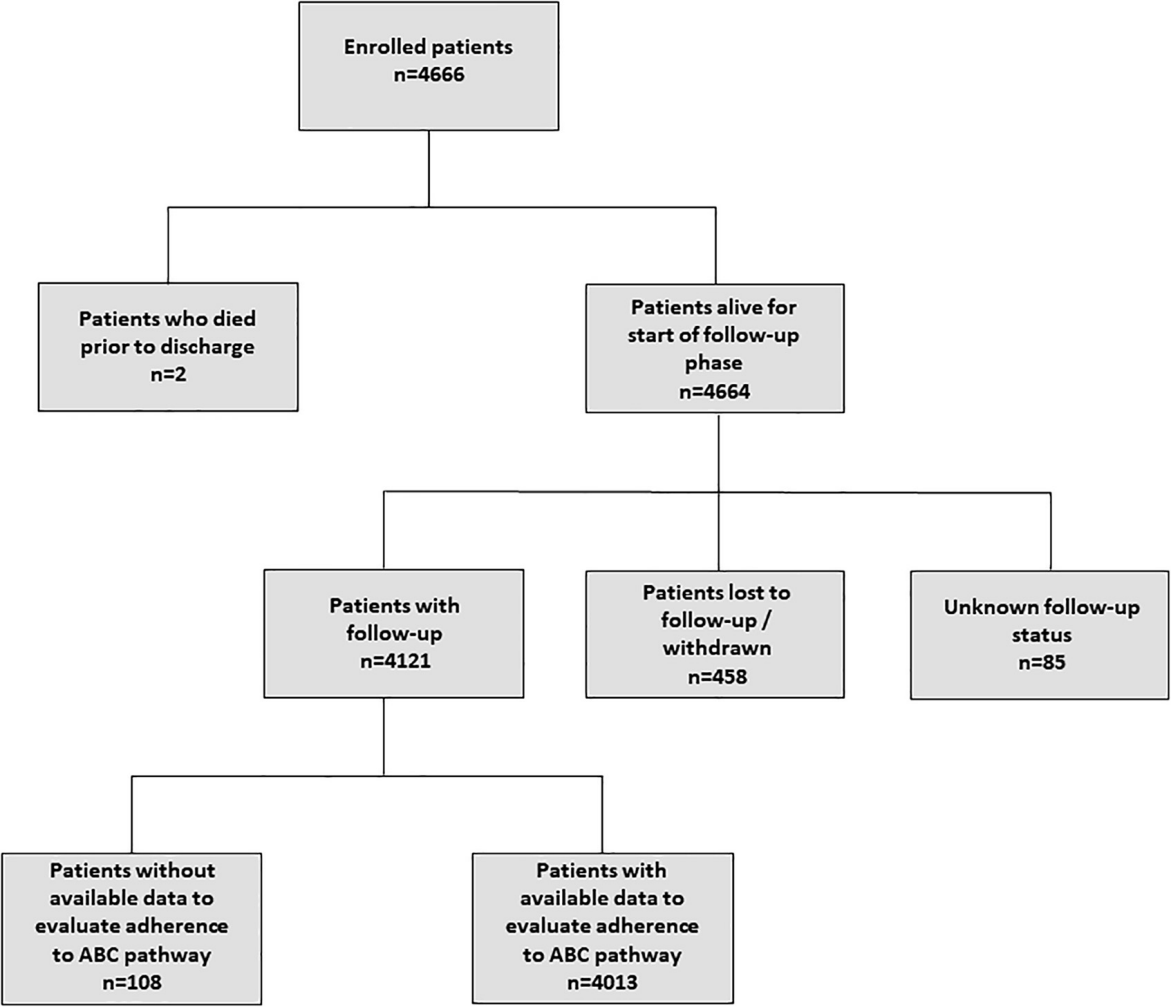
Patients Flow of the Study The APHRS Registry was started in 2015, and the enrollment was finished in 2017. The population comprised consecutive patients with atrial fibrillation who had undergone a cardiology examination in tertiary and general hospitals in 5 Asian-Pacific geographic areas. Among the 4,666 patients enrolled in the APHRS registry, 4,013 (86.1%) had available follow-up data and information needed to evaluate adherence to the ABC pathway. APHRS = Asia-Pacific Heart Rhythm Society.

Among the 4,666 patients enrolled in the APHRS registry, 4,013 (86.1%) had available follow-up data and information needed to evaluate adherence to the ABC pathway (Figure 1). Patients excluded from the analysis were older and had lower CHA_2_DS_2_-VASc and HAS-BLED scores than those included.

### Clinical characteristics

The final study cohort comprised 4,013 patients, of whom 2,464 (61.4%) were ABC nonadherent and 1,549 (38.6%) ABC adherent. As reported in Table 1, ABC nonadherent patients were older and had higher CHA_2_DS_2_-VASc and HAS-BLED scores than ABC- adherent patients.

**Table 1:** Baseline Characteristics of ABC-Adherent and Nonadherent Patients

	ABC Nonadherent (n = 2,464)	ABC Adherent (n = 1,549)	*P* Value
Age (y)	70.2 ± 11.5	65.8 ± 11.9	<0.001
Female	868 (35.2)	511 (33.0)	0.146
AF pattern			
First diagnosed	193 (7.9)	97 (6.3)	<0.001
Paroxysmal	1,003 (40.9)	675 (43.7)	
Persistent	540 (22.0)	421 (27.2)	
Longstanding persistent	246 (10.0)	142 (9.2)	
Permanent	473 (19.3)	211 (13.6)	
Concomitant disease			
Hypertension	1,660 (67.8)	792 (51.3)	<0.001
CAD	670 (27.6)	112 (7.2)	<0.001
HF	692 (28.4)	159 (10.3)	<0.001
Diabetes	740 (30.4)	246 (15.9)	<0.001
Lipid disorder	1,004 (41.4)	519 (33.9)	<0.001
Smoker	204 (8.3)	136 (8.8)	0.579
Previous stroke/TIA	297 (12.2)	90 (5.8)	<0.001
Previous bleedings	233 (9.5)	67 (4.3)	<0.001
ICH	52 (2.1)	16 (1.0)	0.010
Major extracranial bleeding	99 (4.0)	26 (1.7)	<0.001
PAD	43 (1.8)	5 (0.3)	<0.001
CKD	219 (8.9)	90 (5.8)	<0.001
COPD	79 (3.2)	30 (1.9)	0.016
Cancer	68 (2.8)	25 (1.6)	0.019
Dementia	60 (2.4)	9 (0.6)	<0.001
Anemia	225 (9.2)	65 (4.2)	<0.001
Symptomatic status			
EHRA I	1523 (61.8)	1049 (67.7)	<0.001
EHRA II	684 (27.8)	500 (32.3)	
EHRA III	228 (9.3)	0 (0.0)	
EHRA IV	29 (1.2)	0 (0.0)	
Thrombotic and hemorrhagic risk			
CHA_2_DS_2_-VASC score	3.1 ± 1.7	2.1 ± 1.5	<0.001
HAS-BLED	1.5 ± 1.1	1.1 ± 0.9	<0.001

Values are mean ± SD or n (%).

AF = atrial fibrillation; CAD = coronary artery disease; CKD = chronic kidney disease; COPD = chronic obstructive pulmonary disease; EHRA = European Heart Rhythm Association; HF = heart failure; ICH = intracranial hemorrhage; PAD = peripheral artery disease; TIA = transient ischemic attack.

**Figure 2:**
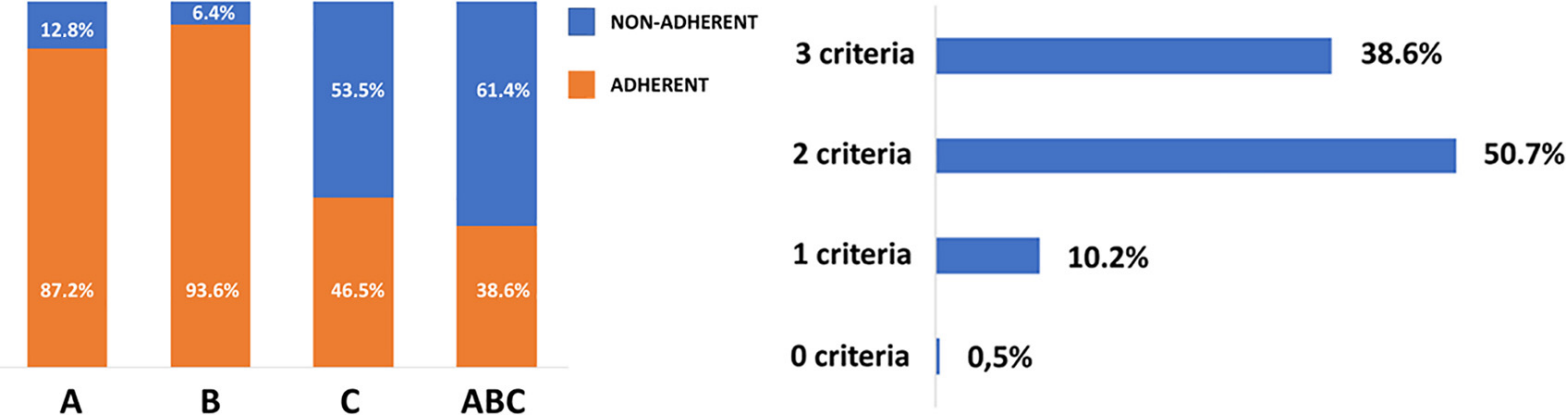
ABC Criteria Adherence and Distribution The ABC pathway has 3 main pillars: “A” for avoiding stroke with oral anticoagulation; “B” refers to symptom control; and “C” for management of cardiovascular risk factors. The patient was considered compliant for the “A” criterion if properly prescribed with oral anticoagulation according to the CHA_2_DS_2_-VASc score. Any patient with no symptoms or with mild symptoms not affecting daily life was qualified for the “B” criterion. A patient was considered adherent to the “C”" criterion when hypertension, coronary artery disease, peripheral artery disease, heart failure, stroke or transient ischemic attack, and diabetes mellitus were treated according to the current clinical guidelines. All patients with ≥1 clinical condition not properly treated were considered to be “C”" criterion nonadherent. Patients were considered treated as adherent to the ABC pathway if they were adherent to all 3 ABC pillars.

Overall, 3,502 patients (87.2%) were adherent to the “A” criterion; 3,756 (93.6%) were adherent to the “B” criterion; and 1,866 (46.5%) were adherent to the “C” criterion. The number of patients with 0, 1, 2, or 3 criteria attained is reported in Figure 2.

### Follow-up and survival analysis

**Figure 3:**
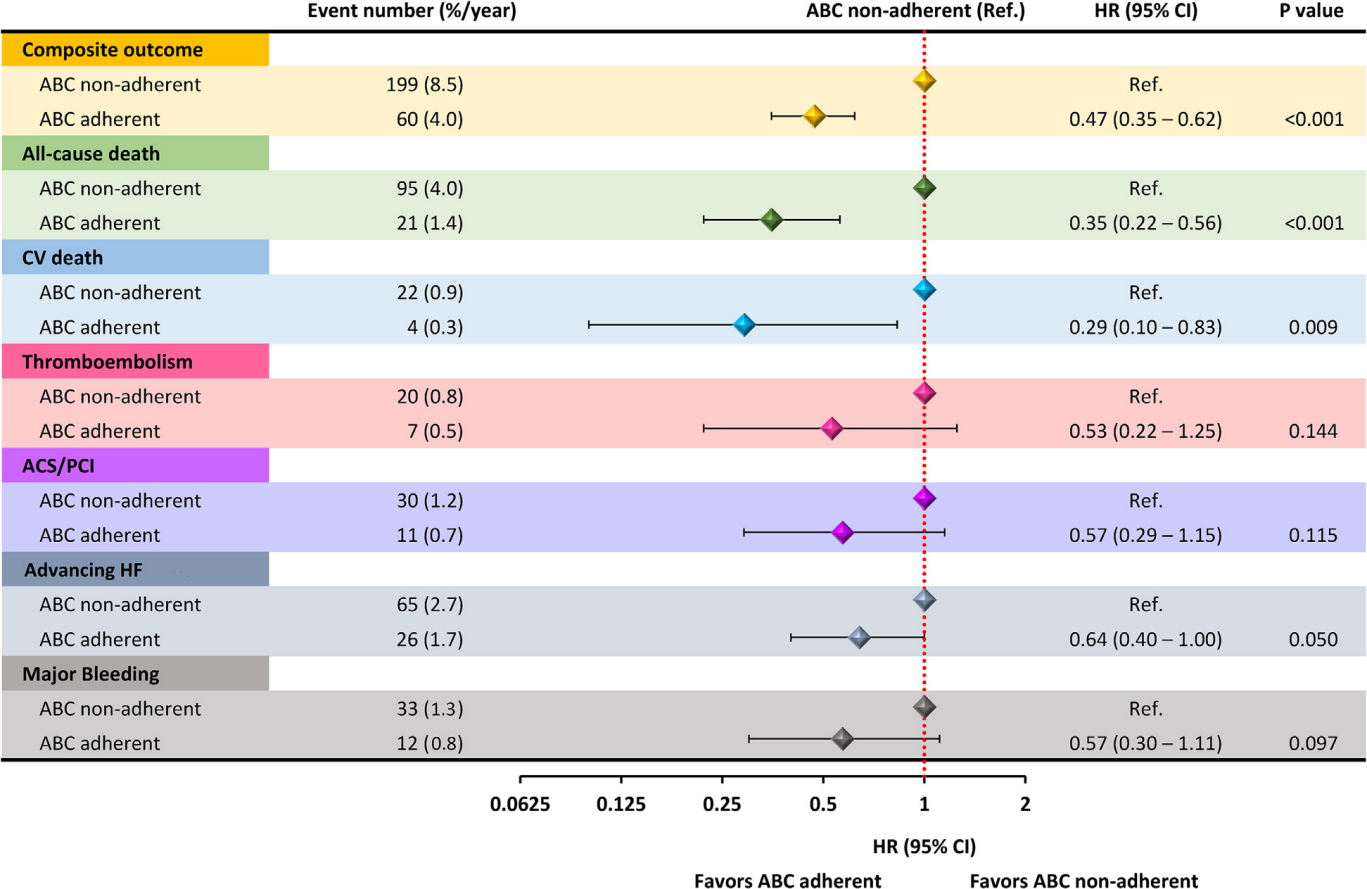
Risk for Primary and Secondary Outcomes on Cox-Regression Univariate Analysis On univariate Cox-regression analysis, ABC-adherent patients showed a lower risk of the composite outcome, all-cause death, and cardiovascular death. No significant associations were found between ABC adherence and the risk of thromboembolism, ACS/PCI, advancing HF, and major bleeding. ACS/PCI = acute coronary syndrome/percutaneous coronary intervention; HF = heart failure.

After 1-year follow-up time, the following events were recorded: 259 (6.7%) composite outcome events, of which 116 (2.9%) were all-cause death, 26 (0.6%) cardiovascular death, 27 (0.7%) any TE, 41 (1.1%) ACS or PCI, 91 (2.3%) advancing HF, and 45 (1.2%) major bleedings. Event rates recorded throughout the follow-up observation according to the adherence to the ABC pathway showed consistently lower incidence rates for patients managed adherent to the ABC pathway for the composite outcome (*P* < 0.001), as well as for all-cause death (*P* < 0.001), cardiovascular death (*P* < 0.001), and advancing HF (*P* = 0.010), but not for the other secondary outcomes (Figure 3).

On univariable Cox-regression analysis, full adherence to the ABC pathway criteria was associated with a lower risk of composite outcome (HR: 0.47; 95% CI: 0.35-0.62), all-cause death (HR: 0.35; 95% CI: 0.22-0.56), and cardiovascular death (HR: 0.29; 95% CI: 0.10-0.83). No significant associations were found between ABC adherence and the other secondary outcomes (Figure 3).

On multivariable Cox-regression analysis adjusted for age, sex, CHA_2_DS_2_-VASc score, paroxysmal AF (vs nonparoxysmal), chronic obstructive pulmonary disease, chronic kidney disease, cancer, dyslipidemia, and dementia, ABC pathway adherence was associated with a lower risk for the composite outcome (HR: 0.72; 95% CI: 0.53-0.97) compared with ABC nonadherent patients (Table 2, Central Illustration A). Using the same multivariable model for the secondary outcomes, we found nonstatistically significant trends in the association of ABC pathway adherence with lower risk of all-cause death (HR: 0.68, 95% CI: 0.41-1.12), cardiovascular death (HR: 0.45; 95% CI: 0.13-1.54), any TE (HR: 0.65; 95% CI: 0.27-1.60), ACS or PCI (HR: 0.68; 95% CI: 0.32-1.43), advancing HF (HR: 0.97; 95% CI: 0.60-1.55), and major bleeding (HR: 0.77; 95% CI: 0.39-1.53).

**Table 2:** Multivariable Cox-Regression Analysis for Occurrence of the Composite Outcome

	HR	95% CI	*P* Value
Age	1.03	1.01-1.04	<0.001
Female	0.86	0.66-1.14	0.299
CHA_2_DS_2_-VASc	1.30	1.18-1.43	<0.001
Paroxysmal AF	0.67	0.51-0.88	0.004
COPD	2.96	1.91-4.63	<0.001
CKD	1.89	1.38-2.59	<0.001
Cancer	2.01	1.22-3.31	0.006
Dyslipidemia	1.47	1.13-1.90	0.004
Dementia	1.51	0.93-2.65	0.090
ABC adherence	0.72	0.53-0.97	0.033

Abbreviations as in Table 1.

**Central illustration:**
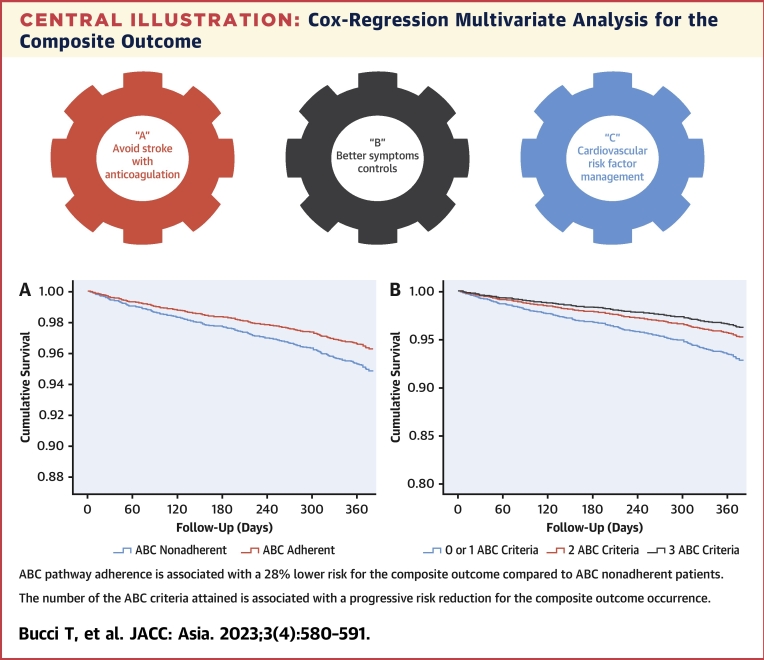
Cox-Regression Multivariate Analysis for the Composite Outcome **(A)** The risk of composite outcome in patients ABC adherent compared with patients ABC nonadherent. **(B)** The risk of composite outcome in patients with 0 or 1 ABC criteria compared with patients with 2 and 3 ABC criteria. The multivariate analysis was adjusted for age, sex, CHA_2_DS_2_-VASc, paroxysmal-AF, chronic obstructive pulmonary disease, chronic kidney disease, cancer, dyslipidemia, and dementia. AF = atrial fibrillation.

An increasing number of the ABC criteria attained was associated with a progressive risk reduction for the composite outcome occurrence. Compared with patients with no ABC criteria, the risk of composite outcome was progressively lower in patients with a progressively higher number of ABC criteria attained after adjustment for age, sex, CHA_2_DS_2_-VASc, paroxysmal AF, chronic obstructive pulmonary disease, chronic kidney disease, cancer, dyslipidemia, and dementia (Table 3, Supplemental Figure 1). Given the small number of patients with no fulfilled ABC criteria, we also repeated this analysis, using—as a reference—the group of patients with 0 or 1 criteria attained. This analysis consistently confirmed that a progressively higher number of criteria attained was associated with a progressively lower risk of the composite outcome (Table 3, Central Illustration B).

**Table 3:** Multivariable Cox-Regression Analysis for the Occurrence of the Composite Outcome (Number of ABC Criteria)

	Model A				Model B		
	HR	95% CI	*P* Value		HR	95% CI	*P* Value
Age	1.03	1.01-1.04	0.001	Age	1.03	1.01-1.04	0.001
Female	0.85	0.64-1.11	0.232	Female	0.85	0.64-1.12	0.248
CHA_2_DS_2_-VASc	1.31	1.19-1.45	<0.001	CHA_2_DS_2_-VASc	1.31	1.18-1.44	<0.001
Paroxysmal AF	0.67	0.51-0.88	0.004	Paroxysmal AF	0.66	0.50-0.86	0.002
COPD	2.87	1.84-4.48	<0.001	COPD	2.86	1.83-4.46	<0.001
CKD	1.80	1.31-2.47	<0.001	CKD	1.82	1.32-2.49	<0.001
Cancer	1.80	1.09-2.97	0.022	Cancer	1.92	1.15-3.21	0.014
Dyslipidemia	1.42	1.10-1.84	0.008	Dyslipidemia	1.43	1.11-1.86	0.006
Dementia	1.59	0.94-2.68	0.082	Dementia	1.58	0.94-2.67	0.085
0 criteria ABC	Ref	-	-	0-1 criteria ABC	Ref.	-	-
1 criterion ABC	0.24	0.11-0.50	<0.001				
2 criteria ABC	0.17	0.08-0.36	<0.001	2 criteria ABC	0.66	0.48-0.91	0.011
3 criteria ABC	0.14	0.07-0.29	<0.001	3 criteria ABC	0.52	0.35-0.76	0.001

Abbreviations as in Table 1.

### Sensitivity analysis

In our study, low-risk patients with no OAC indication, and therefore with no OAC prescription, were considered as adherent to the “A” pillar. To evaluate if the presence of patients with low baseline thromboembolic risk could have influenced the association between adherence to the ABC pathway and the risk of the composite outcome, we performed a sensitivity analysis, considering only patients with CHA_2_DS_2_-VASc ≥2. The cohort selected comprised 2,939 patients, of whom in 1,981 (67.4%) were ABC nonadherent and 958 (32.6%) were ABC adherent. Overall, 2,556 (87.0%) were adherent to the “A” criterion; 2,764 (94.0%) were adherent to the “B” criterion; and 1,137 (38.7%) were adherent to the “C” criterion. The number of patients with respectively 0, 1, 2, or 3 fulfilled criteria was 21 (0.7%), 337 (11.5%), 1,623 (55.2%,), and 958 (32.6%).

Similar to the main analysis, patients managed as adherent to the ABC pathway, compared with those not adherent, reported a lower incidence of the composite outcome (*P* < 0.001), all-cause death (*P* < 0.001), and cardiovascular death (*P* = 0.05) but not for the other secondary outcomes (Supplemental Table 1).

At univariable Cox regression analysis, clinical management adherent to the ABC pathway was associated with a lower risk of composite outcome (HR: 0.51; 95% CI: 0.37-0.71) and all-cause death (HR: 0.47; 95% CI: 0.29-0.75), whereas no significative association was found for cardiovascular death and the other secondary outcomes (Supplemental Table 1).

At multivariable Cox regression analysis, adherence to the ABC pathway was associated with lower risk of composite outcome (HR: 0.68; 95% CI: 0.49-0.95) compared with the ABC nonadherent group (Supplemental Table 2) as well as associated with a progressively lower risk according to a progressively higher number of ABC criteria attained (1 criterion, HR: 0.23; 95% CI: 0.11-0.50; 2 criteria, HR: 0.16; 95% CI: 0.06-0.27; 3 criteria, HR: 0.12; 95% CI: 0.06-0.27) (Supplemental Table 3). Repeating this analysis, using as reference the group of patients with 0 or 1 criterion attained, we confirmed again that a higher number of criteria attained was associated with a progressively lower risk of the composite outcome (HR: 0.63; 95% CI: 0.45-0.87 and HR: 0.47; 95% CI: 0.31-0.71, respectively, for 2 and 3 criteria attained) (Supplemental Table 3).

### Subgroup analysis

**Figure 4:**
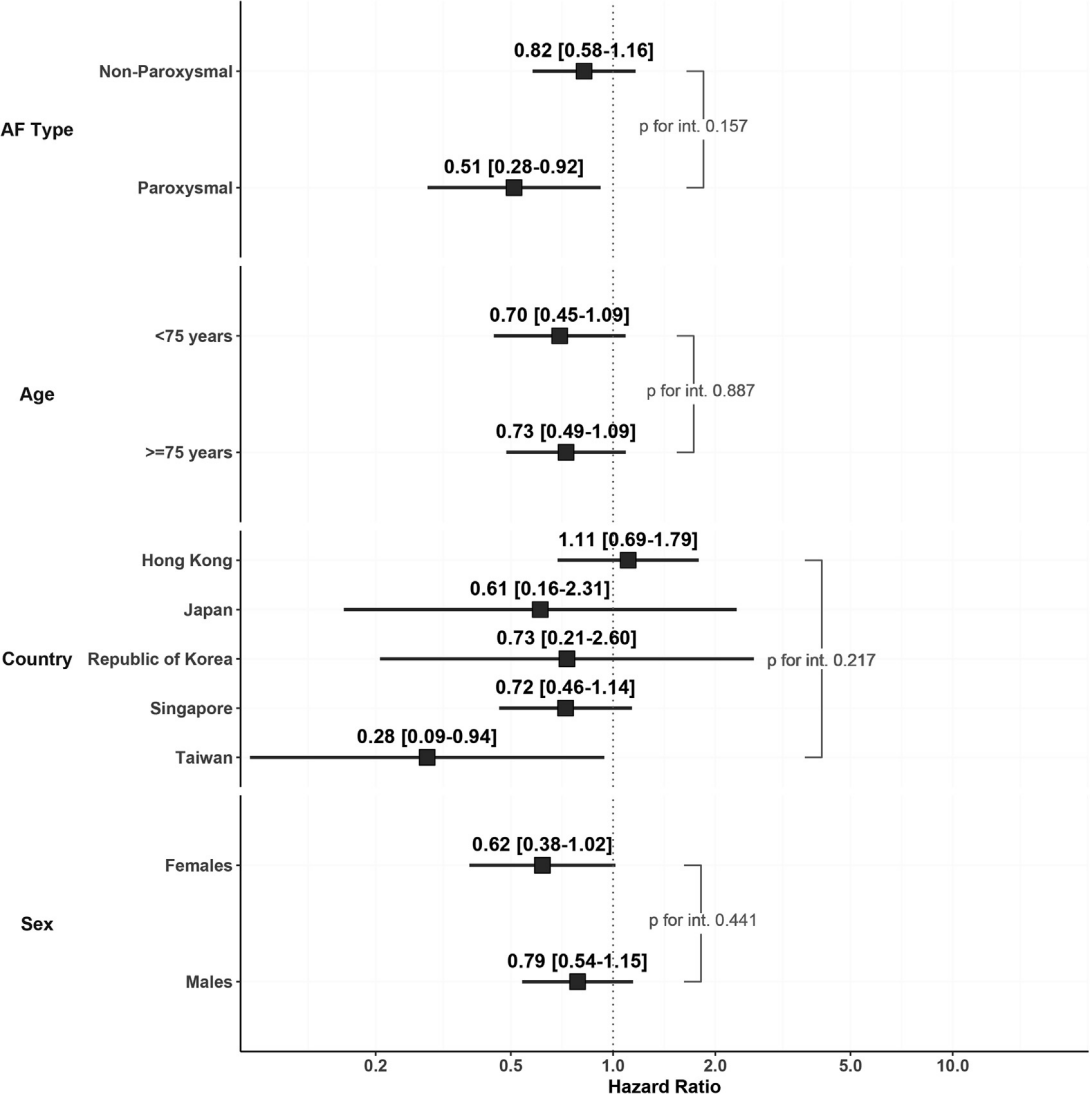
Risk of Composite Outcome in Different Subgroups On Cox regression multivariate analysis adjusted for age, sex, CHA_2_DS_2_-VASc, paroxysmal-AF, chronic obstructive pulmonary disease, chronic kidney disease, cancer, dyslipidemia, and dementia, full adherence to the ABC pathway was associated with a lower risk of the composite outcome irrespective of the origin country, age >75 years, sex, and AF type. AF = atrial fibrillation.

Adherence to ABC was associated with a lower risk of the composite outcome irrespective of the origin country (Figure 4). Similarly, no difference was found in the association between adherence to the ABC pathway and risk of composite outcome according to the clinical subgroups examined (Figure 4).

## Discussion

In this prospective cohort of Asian patients with AF derived from the APHRS Registry, we found that 38.6% of patients showed a full adherence to the ABC pathway management, as they were complying with all 3 main pillars of this integrated approach. Second, ABC pathway adherence was associated with a 28% lower risk of a composite outcome of all-cause death and CVEs, and the risk reduction was proportionally higher according to a higher number of ABC criteria. Third, the association between ABC pathway adherence and lower risk of outcomes was found irrespective of the enrolling country, age, sex, and AF type.

The 38.6% prevalence of the ABC pathway adherence in our population was similar to the 41.1% previously reported from the ChiOTEAF (Optimal Thromboprophylaxis in Elderly Chinese Patients with Atrial Fibrillation) Registry on 3,520 Chinese patients but higher than the 22.3% and 15.3% reported from other 2 studies performed on the Chinese Atrial Fibrillation (CAFR) dataset on 19,187 Chinese patients and on the Korea National Health Insurance Service database on 204,824 Korean patients, respectively.^11^, ^12^, ^13^ These differences in ABC pathway prevalence could be due to the differences in the study design and the characteristics of the patients enrolled. Indeed, although patients enrolled in the ChiOTEAF Registry had similar mean age and even higher CHA_2_DS_2_-VASc scores,^13^ those enrolled in the other 2 studies were largely unselected patients, with lower mean age and lower baseline thromboembolic risk.^11^^,^^12^

Conversely, the prevalence of ABC pathway adherence in our cohort was significantly higher when compared with that reported in a recent meta-analysis (21%).^22^ Of note, the latter meta-analysis was based mainly on non-Asian studies, with higher mean age and baseline thromboembolic risk.

Moreover, we found that in the APHRS Registry, the prevalence of ABC criteria attained was 0.5% for 0 criteria, 10.2% for 1 criterion, 50.7% for 2 criteria, and 38.6% for 3 criteria: a prevalence similar to that reported in the ChiOTEAF registry.^12^ Despite that in our population, adherence for “A” and “B” criteria was approximately 90%, showing good use of OAC and control of symptoms, we found that the “C” criteria were attained in <50% of patients, significantly affecting the overall prevalence of adherence to the ABC pathway.

AF is a clinical condition that often coexists with several diseases that increase the global risk of death and CVEs.^6^ The importance of treating these clinical conditions with the optimal therapies is underlined by the risk reduction of 28% for the composite outcome that we found in our study among those patients who were ABC-pathway adherent. This result is similar to that found in previous studies performed in Asian populations that reported a risk reduction for similar composite outcomes between 24% and 49%^11^, ^12^, ^13^ but is lower than the 40% to 60% risk reduction reported in studies performed in Western countries with higher ABC pathway adherence.^9^^,^^10^^,^^14^, ^15^, ^16^

In our analysis, the lower risk for the composite outcome in the ABC-adherent group seems to be mainly related to the lower risk of all-cause death and cardiovascular death, whereas the weight of the other secondary outcomes appears negligible. When interpreting these results, we must consider the low incidence of cardiovascular events and major bleeding reported, the small sample size, and the short follow-up that could be responsible for limited statistical power. Thus, further studies with larger populations and longer observation times are needed better to investigate these observations.

We also showed a progressive reduction of the risk for the composite outcome according to the increasing ABC criteria attained. This evidence is consistent with a recent paper examining the impact of adherence to the ABC pathway in a large multinational observational AF cohort.^23^ On subgroup analyses, we also observed a similar effect size in patients with age ≥75 or <75 years, female or male patients, with paroxysmal or not paroxysmal AF, as well as in the different Asian countries contributing to the APHRS Registry. Thus, ABC pathway adherence was evident irrespective of enrolling centers (Hong Kong, South Korea, Japan, Singapore, and Taiwan) and some key clinical characteristics.

When considering patients with a low number of cardiovascular comorbidities, the ABC pathway could introduce some bias associated with considering “well-treated” patients with no indication to OAC or no concomitant comorbidities. Therefore, to avoid an overestimation of the protective effect of the ABC pathway adherence in reducing the risk of composite outcome, we performed a sensitivity analysis considering only patients at high risk for TE as indicated by a CHA_2_DS_2_-VASc ≥2. In this analysis, we confirmed the results related to the overall cohort, showing a consistent association with lower risk for composite outcome in ABC-adherent ABC patients and a progressively lower risk for a progressively higher number of ABC criteria attained. These results reinforce the strength of the overall evidence provided by this paper, supporting the pivotal role of this tailored and holistic treatment in reducing the risk of outcomes also in patients with AF at high risk for CVEs.

### Study strengths

These data confirm the benefit of ABC pathway management in patients with AF and reinforce the recommendation for a broader application of ABC management in Asians. Indeed, the ABC pathway is now recommended in international guidelines, including those from the APHRS.^24^ This work is also crucial in the light of the ethnic differences regarding stroke and bleeding risk between Asian and non-Asian patients.^25^^,^^26^

### Study limitations

The observational nature of this study limits the strength and the generalizability of the evidence derived. For the “A” criterion, adherence was considered only if the patient were on OAC; no further evaluation about dosage for DOAC or time in therapeutic range for warfarin users was possible for the lack of this information, which could have provided a more accurate classification of patients. Although we considered approximately 80% of the initial cohort, the small clinical differences among patients included and excluded from this analysis could have introduced some selection bias. Furthermore, the lower prevalence of cardiovascular risk factors found in patients who were ABC adherent compared with those who were ABC nonadherent may have significantly affected the probability of attaining the “C” criteria and the risk of clinical outcomes at 1-year follow-up. Other possible confounding factors, such as smoking habits; physical activity; alcohol consumption; cognitive function; and the optimal management of chronic obstructive pulmonary disease, chronic kidney disease, and dyslipidemia were not considered in this analysis, as specific data about the treatment of these conditions were not collected in the original case report form.

## Conclusions

In a large contemporary cohort of Asian patients with AF, ABC pathway adherence was associated with a reduction of the risk for the composite outcome of all-cause death and CVEs. The beneficial effect of this integrated approach was found irrespective of enrolling country and various clinical characteristics.

Perspectives **COMPETENCY IN PATIENT CARE AND PROCEDURAL SKILLS:** The association between ABC pathway adherence and the reduction of adverse events in the APHRS registry reinforces the recommendation for a broader application of ABC-pathway management in Asians.

**TRANSLATIONAL OUTLOOK:** The concept of a holistic or integrated care management of patients with AF should be improved by promoting adherence to the ABC pathway (“Simple as ABC…!”).

## Funding Support and Author Disclosures

This study was an independent research grant by Pfizer and Bristol Myers Squibb to Asia-Pacific Heart Rhythm Society. This research was partially funded by the Italian Ministry of Health, regarding the work and the role in the paper of Dr Proietti. Dr Proietti is national leader of the AFFIRMO project on multimorbidity in atrial fibrillation, which has received funding from the European Union’s Horizon 2020 research and innovation program under grant agreement No 899871. Dr Romiti has received consultancy fees from Boehringer Ingelheim and an educational grant from Anthos, outside of the submitted work; no fees were directly received personally. Dr Shimizu has received grants from Daiichi Sankyo Co, Ltd and Nippon Boehringer Ingelheim Co, Ltd; and remuneration for lectures, presentations, Speakers Bureau, manuscript writing, or educational events from Daiichi Sankyo Co, Ltd, Nippon Boehringer Ingelheim Co, Ltd, and Bristol Myers Squibb. Bayer Yakuhin, Ltd, Pfizer Japan, Inc, Ono Pharmaceutical Co, Ltd, and Medtronic Japan Co, Ltd. Dr Tse has received consultant and speaker fees and research grants from Abbott, Amgen, AstraZeneca, Bayer, Bristol Myers Squibb, Boehringer Ingelheim, Boston Scientific, Daiichi Sankyo, Medtronic, Novartis, Pfizer, and Sanofi. Dr Lip is a consultant and speaker for Bristol Myers Squibb/Pfizer, Boehringer Ingelheim, Anthos, and Daiichi-Sankyo; has received no fees personally. Dr Lip is coprincipal investigator of the AFFIRMO project on multimorbidity in AF, which has received funding from the European Union’s Horizon 2020 research and innovation program under grant agreement No 899871. All other authors have reported that they have no relationships relevant to the contents of this paper to disclose.
