# Supplementary material for: Integrated Care for Atrial Fibrillation Using the ABC Pathway in the Prospective APHRS-AF Registry
Source: JACC Asia. 2023 Jun 27;3(4):580–91. doi: 10.1016/j.jacasi.2023.04.008 (PMC10442886; doi:10.1016/j.jacasi.2023.04.008)
Supplement: Supplemental Appendix [file mmc1.docx]

**Supplemental Table 1.** Incidence rates and Cox regression analyses for risk of primary and secondary outcomes in patients with CHA_2_DS_2_-VASc≥2 according to ABC adherence.

|  | **Number of events** | **Incidence rate**  **(100 patients/year)** | **Univariate**  **HR (95% CI)** | **p value** | **Multivariate**  **HR (95% CI)** | **p value** |
| --- | --- | --- | --- | --- | --- | --- |
| **Composite outcome** |  |  |  |  |  |  |
| ABC non-adherent | 188 | 10.0 | Ref. | - | Ref. | - |
| ABC adherent | 48 | 5.2 | 0.51 (0.37-0.71) | <0.001 | 0.68 (0.49 – 0.95) | 0.022 |
| **All-cause death** |  |  |  |  |  |  |
| ABC non-adherent | 92 | 4.8 | Ref. | - | Ref. | - |
| ABC adherent | 21 | 2.2 | 0.47 (0.29-0.75) | 0.002 | 0.74 (0.45 – 1.22) | 0.235 |
| **CV death** |  |  |  |  |  |  |
| ABC non-adherent | 22 | 1.1 | Ref. | - | Ref. | - |
| ABC adherent | 4 | 0.4 | 0.37 (0.13-1.08) | 0.068 | 0.45 (0.13 – 1.54) | 0.202 |
| **Thromboembolism** |  |  |  |  |  |  |
| ABC non-adherent | 18 | 0.9 | Ref. | - | Ref. | - |
| ABC adherent | 4 | 0.6 | 0.43 (0.15 – 1.28) | 0.130 | 0.50 (0.17 – 1.53) | 0.228 |
| **ACS/PCI** |  |  |  |  |  |  |
| ABC non-adherent | 28 | 1.2 | Ref. | - | Ref. | - |
| ABC adherent | 9 | 0.7 | 0.66 (0.31 – 1.40) | 0.276 | 0.66 (0.30 – 1.47) | 0.308 |
| **New/worsening HF** |  |  |  |  |  |  |
| ABC non-adherent | 60 | 2.7 | Ref. | Ref. | Ref. | - |
| ABC adherent | 19 | 1.7 | 0.66 (0.39 – 1.11) | 0.114 | 0.92 (0.54 – 1.56) | 0.746 |
| **Major Bleeding** |  |  |  |  |  |  |
| ABC non-adherent | 30 | 1.3 | Ref. |  | Ref. | - |
| ABC adherent | 9 | 0.8 | 0.61 (0.29 – 1.29) | 0.200 | 0.73 (0.34 – 1.57) | 0.419 |

**HR:** Hazard Ratio, **CI**: Confidence interval, **CV**: Cardiovascular, **ACS**: Acute Coronary Syndrome, **PCI**: Percutaneous Coronary Intervention, **HF**: Heart Failure.

**Supplemental Table 2.** Multivariable Cox regression analysis for occurrence of the composite outcome in patients with CHA_2_DS_2_-VASc≥2 considering full ABC adherence vs. non-adherence.

|  | **HR** | **95% CI** | **p value** |
| --- | --- | --- | --- |
| **Age** | 1.04 | 1.02-1.05 | <0.001 |
| **Female sex** | 0.87 | 0.66-1.15 | 0.329 |
| **CHA_2_DS_2_-VASc** | 1.30 | 1.17-1.44 | <0.001 |
| **Paroxysmal AF** | 0.71 | 0.54-0.94 | 0.017 |
| **COPD** | 3.05 | 1.95-4.76 | <0.001 |
| **CKD** | 1.79 | 1.29-2.47 | <0.001 |
| **Cancer** | 1.85 | 1.01-3.14 | 0.022 |
| **Dyslipidaemia** | 1.59 | 1.21-2.09 | 0.001 |
| **Dementia** | 1.47 | 0.87-2.49 | 0.148 |
| **ABC adherence** | 0.68 | 0.49-0.95 | 0.022 |
| **HR:** Hazard Ratio, **CI:** Confidence Interval, **COPD**: Chronic Obstructive Pulmonary Disease, **CKD**: Chronic Kidney Disease. | | | |

**Supplemental Table 3.** Multivariable Cox regression analysis for occurrence of the composite outcome in patients with CHA_2_DS_2_-VASc≥2 considering the number of ABC criteria.

|  |  | **Model A** |  |  |  | **Model B** |  |
| --- | --- | --- | --- | --- | --- | --- | --- |
|  | **HR** | **95% CI** | **p value** |  | **HR** | **95% CI** | **p value** |
| **Age** | 1.04 | 1.02-1.05 | <0.001 | **Age** | 1.04 | 1.02-1.05 | <0.001 |
| **Female sex** | 0.84 | 0.64-1.12 | 0.241 | **Female sex** | 0.85 | 0.64-1.13 | 0.264 |
| **CHA_2_DS_2_-VASc** | 1.32 | 1.19-1.47 | <0.001 | **CHA_2_DS_2_-VASc** | 1.31 | 1.18-1.45 | <0.001 |
| **Paroxysmal AF** | 0.70 | 0.54-0.93 | 0.015 | **Paroxysmal AF** | 0.70 | 0.52-0.92 | 0.010 |
| **COPD** | 2.93 | 1.87-4.58 | <0.001 | **COPD** | 2.92 | 1.86-4.57 | <0.001 |
| **CKD** | 1.69 | 1.22-2.34 | 0.002 | **CKD** | 1.70 | 1.23-2.36 | 0.001 |
| **Cancer** | 1.60 | 0.94-2.74 | 0.083 | **Cancer** | 1.69 | 1.00-2.88 | 0.052 |
| **Dyslipidaemia** | 1.53 | 1.16-2.01 | 0.002 | **Dyslipidaemia** | 1.54 | 1.17-2.03 | 0.002 |
| **Dementia** | 1.49 | 0.88-2.52 | 0.136 | **Dementia** | 1.49 | 0.88-2.51 | 0.139 |
| **0 criteria ABC** | - | - | - | **0-1 criteria ABC** | - | - | - |
| **1 criterion ABC** | 0.23 | 0.11-0.50 | <0.001 | **2 criteria ABC** | 0.63 | 0.45-0.87 | 0.005 |
| **2 criteria ABC** | 0.16 | 0.08-0.34 | <0.001 | **3 criteria ABC** | 0.47 | 0.31-0.71 | <0.001 |
| **3 criteria ABC** | 0.12 | 0.06-0.27 | <0.001 |  |  |  |  |

**Legend:** **HR:** Hazard Ratio, **CI:** Confidence Interval, **COPD**: Chronic Obstructive Pulmonary Disease, **CKD**: Chronic Kidney Disease.

**Supplemental Figure 1.** Multivariate Cox-regression analysis for composite outcome according to the number of ABC criteria attained.


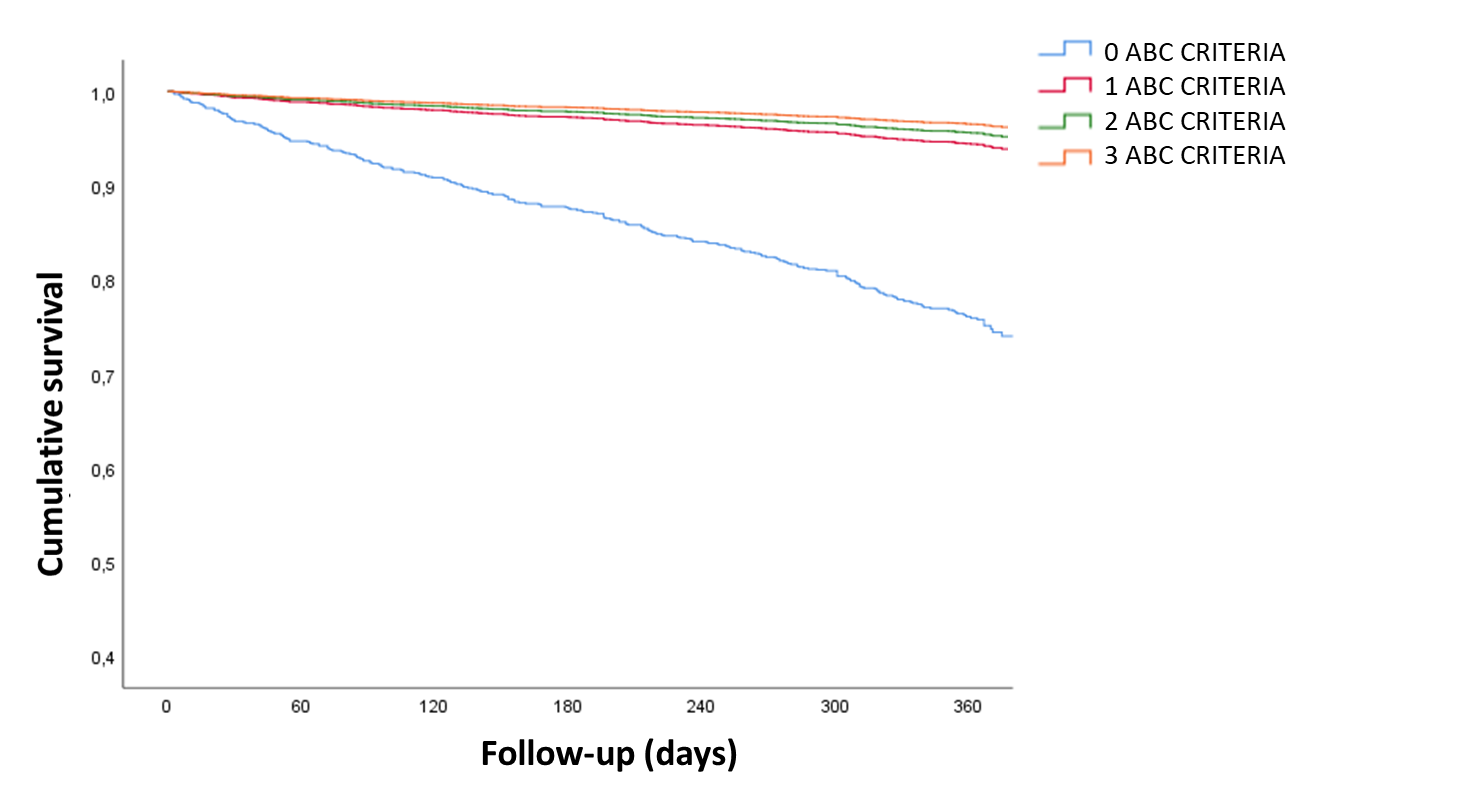


**Asia-Pacific Heart Rhythm Society Atrial Fibrillation Registry Investigators:**

Hong Kong: Chun-Wah Siu David (Queen Mary Hospital).

Japan: Wataru Shimizu, Kenji Yodogawa (Department of Cardiovascular Medicine Graduate School of Medicine Nippon Medical School); Hiroyuki Tsutsui, Yasushi Mukai (Department of Cardiovascular Medicine, Faculty of Medical Sciences, Kyushu University); Hirofumi Tomita, Daisuke Horiuchi (Department of Cardiology, Hirosaki University Graduate School of School of Medicine); Joji Hagii (Hirosaki Stroke and Rehabilitation Center); Kazutaka Aonuma (Division of Cardiology, University of Tsukuba Hospital); Yasuo Okumura (Division of Cardiology, Nihon University Itabashi Hospital); Masahiko Goya, Kenzo Hirao (Department of Cardiovascular Medicine, Tokyo Medical and Dental University); Masayoshi Ajioka (Division of Cardiology, Tosei General Hospital); Nobuhisa Hagiwara, Atsushi Suzuki (Department of Cardiology, Tokyo Women's Medical University); Teiichi Yamane (Department of Cardiovascular Medicine, Jikei University); Takanori Ikeda, Hitomi Yuzawa (Toho University (Faculty of Medicine)); Kazuhiro Satomi, Yoshinao Yazaki (Heart Rhythm Center, Tokyo Medical University); Keiichi Fukuda(Department of Cardiology, Keio University School of Medicine); Yoshinori Kobayashi, Norishige Morita (Division of Cardiology, Tokai University Hachioji-hospital); Toyoaki Murohara (Department of Cardiology, Nagoya University); Eiichi Watanabe, Masahide Harada (Department of Cardiology, Fujita Health University School of Medicine); Satoru Sakagami, Takahiro Saeki (National Hospital Organization Kanazawa Medical Center); Kengo Kusano, Koji Miyamoto (Department of Cardiovascular Medicine, National Cerebral and Cardiovascular Center); Shinsuke Miyazaki, Hiroshi Tada (Department of Cardiovascular Medicine , Faculty of Medical Sciences, University of Fukui); Koichi Inoue, Nobuaki Tanaka(Cardiovascular center, Sakurabashi Watanabe Hospital); Yukihiro Koretsune, Haruhiko Abe (National Hospital Organization Osaka National Hospital, (Osaka, Japan); Yasuki Kihara, Yukiko Nakano (Department of Cardiovascular Medicine, Hiroshima University Graduate School of Biomedical and Health Sciences); Akihiko Shimizu, Yasuhiro Yoshiga (Department of Medicine and Clinical Science、University Graduate School of Medicine); Tomohiro Sakamoto, Ken Okumura(Division of Cardiology, Saiseikai Kumamoto Hospital Cardiovascular Center); Naohiko Takahashi, Tetsuji Shinohara (Oita University Hospital); Kyoko Soejima (Department of Cardiovascular Medicine, Kyorin University School of Medicine); Masahiko Takagi(Kansai Medical University Medical Center); Mitsuharu Kawamura, Yumi Munetsugu (Division of Cardiology, Showa University School of Medicine)

Korea: Sung-Hwan Kim (Division of Cardiology, Department of Internal Medicine, Seoul St. Mary's Hospital, College of Medicine, The Catholic University of Korea, Seoul, South Korea); Jae-Min Shim (Division of Cardiology, Korea University College of Medicine and Korea University Medical Center, Seoul, Republic of Korea); Jae Sun Uhm (Division of Cardiology, Department of Internal Medicine, Yongin Severance Hospital, Yonsei University College of Medicine, Yongin, Korea); Sung Il Im (Division of Cardiology, Department of Internal Medicine, Kosin University Gospel Hospital, Kosin University College of Medicine, Busan, Korea); Hyoung-Seob Par (Division of Cardiology, Department of Internal Medicine, Keimyung University Dongsan Hospital, Daegu, South Korea); Jun Hyung Kim (Department of Cardiology, Chungnam National University, Daejeon, Republic of Korea); Young Keun On (Division of Cardiology, Department of Medicine, Heart Vascular and Stroke Institute, Samsung Medical Center, Sungkyunkwan University School of Medicine); Il-Young Oh (Division of Cardiology, Department of Internal Medicine, Seoul National University Bundang Hospital); Seung Yong Shin (Cardiovascular & Arrhythmia Centre, Chung-Ang University Hospital, Chung-Ang University, Seoul, Korea); Jum Suk Ko (Division of Cardiology, Department of Internal Medicine, Wonkwang University School of Medicine, Iksan, Korea); Jun Beom Park (Department of Cardiology, College of Medicine, Ewha Womans University, Seoul, Korea)

Singapore: Wee-Siong Teo (National Heart Centre Singapore); Kelvin Cheok-Keng Wong (Changi General Hospital); Toon-Wei Lim (National University Hospital); David Foo (Tan Tock Seng Hospital)

Taiwan: Shih-Ann Chen (Taichung Veterans General Hospital); Shih-Ann Chen, Tze-Fan Chao, Yenn-Jiang Lin, Fa-Po Chung, Yu-Feng Hu, Shil-Lin Chang, Ta-Chuan Tuan, Jo-Nan Liao (Taipei Veterans General Hospital); Cheng-Hung Li, Jin-Long Huang, Yu-Cheng Hsieh, Tsu-Juey Wu, Ying-Chieh Liao (Taichung Veterans General Hospital); Cheng-Hung Chiang, Hsiang-Chiang Hsiao, Tung-Chen Yeh (Kaohsiung Veterans General Hospital); Wei-Siang Lin, Wen-Yu Lin (Tri-Service General Hospital); Jen-Yuan Kuo, Chong-Lie Hong, Yih-Je Wu, Ying-Siang Li, Jui-Peng Tsai, Kuo-Tzu Sung, Sheng-Hsiung Chang (Mackay Memorial Hospital).
